# Genetic polymorphisms and protein levels in vocal fold leukoplakia: a systematic review

**DOI:** 10.1590/1414-431X2022e11920

**Published:** 2022-03-11

**Authors:** C.P. Campello, M.F.B. Lima-Silva, E.L.S. de Lima, G.R.S. Nunes, H.A.M. Silva, E. Dellalibera, L.R.P.B. de Britto, C.A.A. Lemos, M.T.C. Muniz

**Affiliations:** 1 Programa Associado de Pós-Graduação em Fonoaudiologia, Universidade Federal da Paraíba, João Pessoa, PB, Brasil; 2 Laboratório de Biologia Molecular, Centro de Oncohematologia Pediátrica, Hospital Universitário Oswaldo Cruz, Universidade de Pernambuco, Recife, PE, Brasil; 3 Programa de Pós-Graduação em Ciências da Saúde, Faculdade de Ciências Médicas, Universidade de Pernambuco, Recife, PE, Brasil; 4 Instituto de Ciências Biológicas, Universidade de Pernambuco, Recife, PE, Brasil; 5 Universidade de Pernambuco, Campus Petrolina, Petrolina, PE, Brasil; 6 Departamento de Odontologia, Universidade Federal de Juiz de Fora, Governador Valadares, MG, Brasil

**Keywords:** Vocal cords, Leukoplakia, Precancerous conditions, Genetic markers, Molecular biology

## Abstract

Vocal fold leukoplakia (VFL) has a risk of malignant transformation. Therefore, patients can have symptoms such as dysphonia, vocal strain, difficulty breathing, and dysphagia. Additionally, there is a genetic predisposition that can be associated with genetic polymorphisms. We aimed to evaluate the influence of genetic polymorphisms and protein levels in the etiology of VFL. Our study followed the PRISMA checklist and was registered on PROSPERO database. The questions were: “Are genetic polymorphisms involved in the etiology of VFL? Are protein levels altered in patients with VFL?”. Eligibility criteria were case control studies that compared the presence of polymorphisms or/and protein levels of subjects diagnosed with VFL and healthy controls. Of the 905 articles retrieved, five articles with a total of 1038 participants were included in this study. The C allele of the single nucleotide polymorphisms (SNP)-819 T/C *IL-10*, A allele of the SNP -592 A/C *IL-10*, CT genotype of the SNP rs11886868 C/T *BCL11A*, GG genotype of the SNP rs4671393 A/G *BCL11A*, LL genotype, and L allele of (GT)n repeat polymorphisms of the *HO-1* were risk factors for VFL development. Nevertheless, there was a lack of association between VFL and the -1082 A/G *IL-10*, rs14024 *CK-1*, and -309 T/G *Mdm2* SNPs. The concentrations of the MDM2, BCL11A, and HO-1 proteins were modified, while IL-10 levels were normally expressed in these subjects. In conclusion, most markers evaluated in this review could be potential indicators to develop effective therapies, avoiding a malignant transformation of the lesion.

## Introduction

Vocal fold leukoplakia (VFL) is a clinical diagnosis of white plaque lesions on the vocal fold epithelial surface (1). This condition may or may not be related to dysplasia (2), and there is a risk of malignant transformation (3). Patients with VFL can have symptoms such as dysphonia, vocal strain, difficulty breathing, and dysphagia (4).

The risk factors for VFL are consumption of alcohol and tobacco, pulmonary disease, diabetes mellitus, hypertension, hyperlipidemia, reflux disease (4), voice abuse (5), and in recent years, some studies have demonstrated that there is a genetic predisposition that can be associated with genetic polymorphisms (6,7). The management of VFL is still controversial because there is no international consensus on a surgical procedure, an effective treatment approach, the frequency of surveillance, and conservative or excisional management (1).

Molecular evaluation of VFL has been indicated to further characterize the lesion (8). Investigations demonstrated that molecular markers such as genetic polymorphisms and protein concentrations are associated with a susceptibility to develop VLF (5,6). The combination of different types of molecular data has indicated the genetic basis of diseases helping define the clinical status of patients (9,10). With this in mind, single nucleotide polymorphisms (SNPs), the most common type of mutation, have been predominant in the study of the link between genetic variations and pathologies (11). SNPs involve the replacement of one nucleotide for another, usually involving the substitution of cytosine (C) for thymine (T) (12). Microsatellite repeats are another type of polymorphism involving 1 to 10 nucleotides (13). They are simple DNA segments that constitute genomic repeat regions (13).

Therefore, it is essential to identify molecular markers that may contribute to the detection of VFL for a better understanding of etiology, pathogenesis, lesion characteristics (1), new diagnostic methods, and treatments strategies (14). Gene-based high-throughput assays that can detect predictive and prognostic gene markers are emerging in healthcare as effective methods to support clinical decision making that may also be applicable in VFL (15).

Studies have found a significant positive association between molecular markers and VFL (6,16), but some researchers did not find a genetic association (5,17). Besides, the lack of review studies on this topic emphasizes the need for evidence synthesis to better understand the genetics in VFL etiology.

In order to have markers for the development of new diagnostic methods and effective treatments to facilitate clinical practice, this systematic review aimed to evaluate the influence of genetic polymorphisms and protein levels on the etiology of VFL. Our research hypotheses were 1) genetic polymorphisms are involved in VFL etiology and 2) proteins levels are altered in VFL.

## Material and Methods

### Protocol registration

The present study followed the Preferred Reporting Items for Systematic Reviews and Meta-Analyses (PRISMA) checklist (18) and was structured based on models published in the literature (19-21). Moreover, the review protocol was registered in the International Prospective Register of Systematic Reviews, PROSPERO (CRD number 42020219983).

### Eligibility criteria

Two questions were addressed in this systematic review, which was based on the Population, Intervention, Comparison, and Outcome (PICO) model. The questions were: “Are genetic polymorphisms involved in the etiology of vocal fold leukoplakia? Are protein levels altered in patients with vocal fold leukoplakia?”. Thus, the Population was participants diagnosed with VFL and healthy controls; the Intervention/Exposure were polymorphisms and measurement of protein concentrations in participants with VFL; and the Comparison was with healthy individuals. The primary Outcome was VFL according to polymorphisms or not and the secondary Outcome was modified protein levels in patients with VFL or not.

Inclusion criteria were studies published in English that compared the presence of polymorphisms or/and proteins between subjects diagnosed with VFL and healthy controls. Exclusion criteria were case reports, reviews, and articles with other molecular markers.

### Search methods

On January 4, 2021, C.P. Campello and C.A.A. Lemos, two independent researchers, performed an online search of PubMed/MEDLINE, The Cochrane Library, Web of Science, and Embase databases for articles published in December 2020 or earlier that met the eligibility criteria. In addition, Open Grey (www.opengrey.eu) was accessed to consult the gray literature. The search terms were: “Vocal Cords Leukoplakia OR Vocal Cord Dysfunction Leukoplakia OR Vocal Fold Leukoplakia OR Vocal Cords Genetic Markers OR Vocal Cord Dysfunction Genetic Markers OR Vocal Fold Genetic Markers OR Vocal Cords Polymorphism OR Vocal Cord Dysfunction Polymorphism OR Vocal Fold Polymorphism OR Vocal Cords Interleukin OR Vocal Cord Dysfunction Interleukin OR Vocal Fold Interleukin” in combination with the Boolean operator.

The authors (C.P.C. and C.A.A.L.) read all the titles and abstracts. When data in the title and abstract were not enough to make a decision, the whole study was acquired. Articles were excluded when they failed to meet the eligibility criteria.

### Data collection process

One investigator (C.P.C.) extracted the data from the studies, a second author (C.A.A.L.) revised all the data collected, and a third author (M.T.C. Muniz) evaluated the divergences in the selection between the researchers. In this way, agreement was achieved. The researchers collected variables such as author, type of study, number of subjects with VFL, number of healthy individuals, mean age, gender, the presence of polymorphisms, and protein concentrations.

### Quality assessment of included studies

The risk of bias of selected studies was evaluated using the Newcastle-Ottawa Scale (NOS) (22), which is based on blinding, outcome data, and other possible biases. The appraisal is based on the selection of study groups, their comparability, and the investigation of exposure. The NOS uses eight questions that evaluate the quality of studies. A maximum of nine stars can be assigned to a study, with a maximum of four stars for selection, two stars for compatibility, and three stars for exposure.

### Additional analysis

The Kappa inter-rater test was used to establish the inter-rater agreement of articles selected in PubMed/MEDLINE, The Cochrane Library, Web of Science, and Embase databases.

## Results

### Literature search

**Figure 1:**
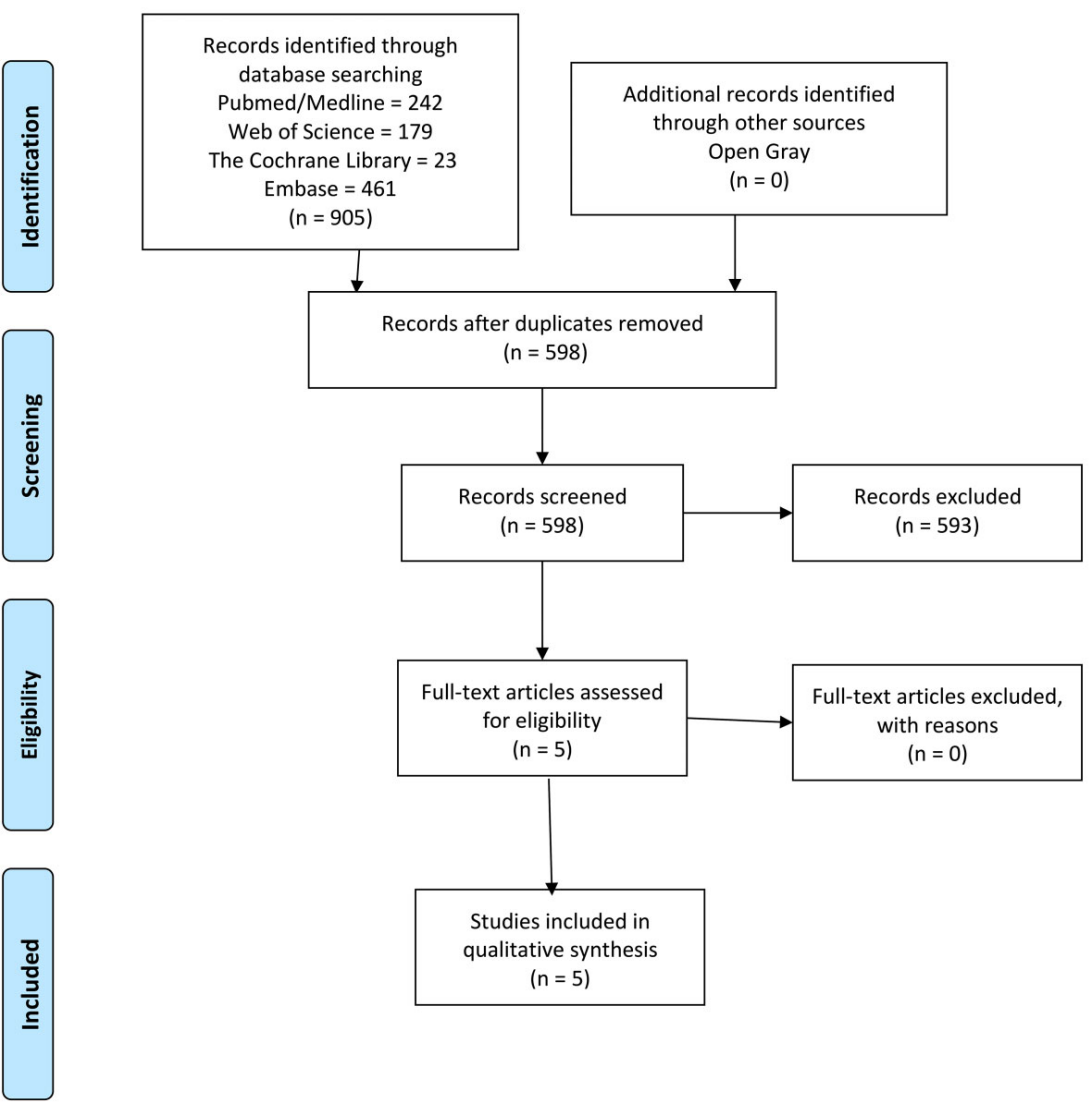
PRISMA flow diagram of study selection.

The details about the article selection process are shown in a flowchart (Figure 1). The search yielded 905 articles: 242 from Pubmed/MEDLINE, 179 from Web of Science, 23 from The Cochrane Library, and 461 from Embase. After duplicate studies were eliminated, 598 articles remained. The titles and abstracts were reviewed considering the inclusion and exclusion criteria. Finally, 5 articles were considered eligible for this systematic review: Zhou et al. (23), Zhou et al. (17), Tang et al. (16), Zhou et al. (6), and Yang et al. (5).

The kappa inter-rater agreement was high (Kappa coefficient=1.00).

### Description of the studies

Details about the five included studies are described in Table 1. All were case-control studies that investigated the presence of polymorphisms or/and protein concentrations in patients with VFL and healthy controls. The findings of these studies were: i) incidence of -1082 A/G, -819 T/C, and -592 A/C interleukin (*IL)-10* SNPs and IL-10 levels (23); ii) occurrence of -309T/G Murine double-minute 2 (*MDM2*) SNP and MDM2 expression (17); iii) presence of (GT)n repeat polymorphisms in the heme oxygenase-1 (*HO-1*) gene and HO-1 concentration (16); iv) presence of rs11886868 C/T and rs4671393 A/G B-cell lymphoma/leukemia 11A (*BCL11A*) SNPs and BCL11A levels (6); and v) detection of the rs14024 cytokeratin 1 (*CK-1*) SNP (5).

**Table 1:** Profile of patients and controls.

Studies on vocal fold leukoplakia	Patients (n)	Controls (n)	Gender		Mean age		Ethnicity
			Patients	Controls	Patients	Controls	
Zhou et al. (23)	61	119	2 females	5 females	56.54±10.7	62.32±7.9	Chinese
			59 males	114 males			
Zhou et al. (17)	61	212	2 females	9 females	56.54±10.7	61.34±6.8	Chinese
			59 males	203 males			
Tang et al. (16)	54	98	3 females	1 female	57.59±9.73	68.32±11.85	Chinese
			51 males	97 males			
Zhou et al. (6)	155	310	5 females	15 females	58.67±7.9	60.37±5.9	Chinese
			150 males	295 males			
Yang et al. (5)	155	266	5 females	8 females	58.63±9.5	61.45±7.7	Chinese
			150 males	258 males			

Data are reported as means±SD.

A total of 1038 participants were included in this systematic review. Three hundred and sixty-four individuals were diagnosed with VFL, 13 females and 351 males. The healthy control group consisted of 674 individuals, 24 females and 650 males.

### Quality assessment and risk of bias of included studies

The studies by Zhou et al. (23), Zhou et al. (17), Tang et al. (16), and Zhou et al. (6) scored seven stars, while the study by Yang et al. (5) scored six stars, which indicated that there was a low risk of bias in all articles. The studies lost a star because they did not report if the controls were from the community and if they had a negative history of VFL. Additionally, the study by Yang et al. (5) lost a star because they analyzed an additional factor in the cases subgroup but did not consider the controls in this analysis (Table 2).

**Table 2:** Risk of bias of case-control studies according to Newcastle-Ottawa Scale.

Studies	Selection	Comparability	Exposure	Total
Zhou et al. (23)	☆☆	☆☆	☆☆☆	7
Zhou et al. (17)	☆☆	☆☆	☆☆☆	7
Tang et al. (16)	☆☆	☆☆	☆☆☆	7
Zhou et al. (6)	☆☆	☆☆	☆☆☆	7
Yang et al. (5)	☆☆	☆	☆☆☆	6

### Presence of SNP and VFL

The presence of SNPs in patients with VFL and healthy controls was analyzed in four studies (Table 3). One of them provided data from three SNPs of the *IL-10* gene, -819 T/C, -592 A/C, and -1082 A/G (23). This study included 61 patients and 119 controls. Regarding the -819 T/C*IL-10* SNP, the cases were 23TT: 27TC: 11CC, while healthy individuals were 64TT: 39TC: 16CC, showing that the TC genotype was a borderline risk factor for developing VFL (OR=1.93, P=0.05). The T allele was present in 73 patients and 167 controls and the C allele was found in 49 patients and 71 healthy subjects, demonstrating that this allele is a risk factor for VFL (OR=1.58; P=0.049).

**Table 3:** Distribution of genotypes and alleles for polymorphisms in cases and controls.

Studies	SNP	VFL patients genotypes/alleles			Controls genotypes/alleles			P	OR
Zhou et al. (23)	*IL-10*	AA	AG	GG	AA	AG	GG	0.092	2.14
	-1082 A/G	50	11	0	107	11	1		
	rs1800896	A	G		A	G		0.201	1.72
		111	11		225	13			
Zhou et al. (23)	*IL-10*	TT	TC	CC	TT	TC	CC	0.05	1.93
	-819 T/C	23	27	11	64	39	16		
	rs1800871	T	C		T	C		0.049	1.58
		73	49		167	71			
Zhou et al. (23)	*IL-10*	AA	AC	CC	AA	AC	CC	0.05	1.93
	-592 A/C	23	27	11	64	39	16		
	rs1800872	A	C		A	C		0.049	1.58
		73	49		167	71			
Zhou et al. (17)	*MDM2*	TT	GT	GG	TT	GT	GG	0.39	0.72
	-309 T/G	13	29	19	35	109	68		
	rs2279744	T	G		T	G		0.57	0.89
		55	67		179	245			
Zhou et al. (6)	*BCL11A*	CC	CT	TT	CC	CT	TT	0.011	3.30
	C/T	144	11	0	302	7	1		
	rs11886868	C	T		C	T		0.038	2.50
		299	11		611	9			
Zhou et al. (6)	*BCL11A*	AA	AG	GG	AA	AG	GG	0.041	3.02
	A/G	4	43	108	19	121	170		
	rs4671393	A	G		A	G		0.002	1.75
		51	259		159	330			
Yang et al. (5)	*CK-1*	AA	AG	GG	AA	AG	GG	0.11 AG	1.82
	rs14024	10	86	59	30	342	94	0.12 GG	1.88
		A	G		A	G		0.96	1.18
		106	330		202	330			

SNP: single nucleotide polymorphism, VFL: vocal fold leukoplakia, OR: odds ratio, IL-10: interleukin 10 gene, MDM2: murine double minute 2 gene, BCL11A: B-cell lymphoma/leukemia 11A gene, CK-1: cytokeratin-1 gene.

Similarly, regarding the -592 A/C *IL-10* SNP, patients had the 23AA: 27AC: 11CC genotypes and controls had the 64AA: 39AC: 16CC genotypes, indicating that the AC genotype was a borderline risk factor for VFL (OR=1.93, P=0.05). The alleles in cases were 49A: 73C, while in controls were 71A: 167C, illustrating that the A allele is a risk factor for VFL (OR=1.58, P=0.049).

On the other hand, the investigation of the -1082 *IL-10* SNP detected 50AA: 11AG: 0GG genotypes in cases and 107AA: 11AG: 1GG in the healthy group (AG; OR=2.14, P=0.09). The alleles were present in the experimental group, A111: G11, and in controls, A225: G13 (OR=1.72, P=0.20), showing no association with VFL.

The second study evaluated the -309 T/G *Mdm2* SNP in 61 patients and 212 healthy people (17). The experimental group presented the 13TT: 29TG: 19GG genotypes and the control group, 35TT: 109GT: 68TT (OR=0.72, P=0.39). Fifty-five patients presented the T allele and 67 the G allele, while the healthy subjects had 179T: 245G (OR=0.89, P=0.57), showing no involvement with VFL etiology.

The third study analyzed two SNPs of the *BCL11A* gene in 155 cases and 310 controls (6). Concerning the rs11886868 C/T *BCL11A* SNP, the CT genotype was frequent in patients (144CC: 11CT: 0TT), but the control group had 302CC: 7CT: 1TT, showing that the CT genotype considerably increased the risk of VFL (OR=3.30, P=0.011). In addition, the T allele was significantly higher in subjects with VFL, 299C: 11T, than in healthy people, 611C: 9T (OR=2.50, P=0.038).

The GG genotype of rs4671393 A/G *BCL11A* SNP was overrepresented in cases (4AA: 43AG: 108GG) compared with controls (19AA: 121AG: 170GG) (OR=3.02, P=0.041). Furthermore, the G allele was a significant risk factor for VFL development, as patients were 51A: 259G while controls were 159A: 461G (OR=1.75, P=0.002).

The fourth study analyzed the rs14024 CK-1 SNP, and 155 VFL subjects had the 10AA: 86AG: 59GG genotypes and 266 healthy people had the 30AA: 142AG: 94GG genotypes, with no statistical difference (AG, OR=1.82, P=0.12; GG, OR=1.88, P=0.11). Similar results can be seen with alleles, with cases being A106: 204G and controls being A202: G330 (OR=1.18, P=0.27) (5).

### Microsatellite repeat polymorphisms and VFL

One study examined the (GT)n repeat polymorphisms in the *HO-1* gene (Table 4), and the LL genotype was significantly more common in individuals with VLF (9LL: 3ML: 29SL: 0MM: 4SM: 9SS) than in controls (5LL: 6ML: 43SL: 3MM: 14SM: 27SS) (OR=3.72, P=0.039). Moreover, the L allele was considerably higher in the patient group (49L: 8M: 51S) than in the control group (58L: 27M: 111S) (OR=1.9, P=0.006), showing that the LL genotype and the L allele are risk factors for VFL (16).

**Table 4:** Microsatellite repeat polymorphisms in cases of vocal fold leukoplakia (VFL) and controls.

Study	(GT)n *HO-1* rs3074372						P	OR
	VFL patients			Controls				
Tang et al. (16)								
Genotypes	LL	ML	SL	LL	ML	SL	0.039	3.72
	9	3	29	5	6	43		
	MM	SM	SS	MM	SM	SS		
	0	4	9	3	14	27		
Alleles	L	S	M	L	S	M	0.006	1.9
	41	42	7	54	84	23		

### Expression of protein levels and VFL

Four studies (6,16,17,23) evaluated protein levels in patients with VFL and controls (Table 5). The proteins MDM2 and BCL11A were overexpressed in the VFL group compared with the control group (P<0.01, P<0.01).The concentration of HO-1 was significantly lower in cases than in controls (P<0.01). Nevertheless, no statistical difference was found between cases and controls concerning IL-10 levels (P>0.05).

**Table 5:** Plasma levels of proteins in patients with vocal fold leukoplakia and control group.

Studies	Protein	Protein detection method	Patients	Controls	P value
Zhou et al. (23)	IL-10	ELISA	20.33±3.1 pg/mL	19.02±7.01 pg/mL	>0.05
Zhou et al. (17)	MDM2	ELISA	301.42±8.6 pg/mL	255.76±8.2 pg/mL	<0.01
Tang et al. (16)	HO-1	ELISA	1.271±0.632 ng/mL	2.069±0.607 ng/mL	<0.01
Zhou et al. (6)	BCL11A	ELISA	80.63 μg/L	71.97 μg/L	<0.01

Data are reported as means±SD. Chi-squared test. IL-10: interleukin 10; MDM2: murine double-minute 2; HO-1: heme oxygenase-1; BCL11A: B-cell lymphoma/leukemia 11A.

## Discussion

This systematic review aimed to investigate the influence of genetic polymorphisms and protein levels in the etiology of VFL for the improvement of diagnostic methods and clinical treatments. This study included articles that evaluated genetic polymorphisms in subjects with VFL comparing their results with healthy individuals. A total of 364 patients were included, 13 females and 351 males. The prevalence of this lesion in males was reported by some studies (4,24-26).

The C allele of the -819 T/C *IL-10* SNP and the A allele of the -592 A/C *IL-10* SNP increased the risk of suffering from VFL (OR=1.58, P=0.049; OR=1.58, P=0.049) (23). Concerning the -1082 A/G *IL-10* SNP, there was a lack of association between this genetic polymorphism and VFL. These three *IL-10* SNPs are located in the promoter region of the gene (27). The T allele of the -819 T/C SNP, the A allele of the -592 A/C *IL-10* SNP, and the A allele of the -1082 A/G *IL-10* SNP were associated with lower IL-10 concentration, while the CCG haplotypes of these SNPs, respectively, were related to higher IL-10 secretion (28).

The normal IL-10 levels in subjects with VFL could be explained by the fact that most of the patients presented the C allele of the -819 T/C*IL-10* SNP correlated to greater IL-10 production and the A allele of -592 A/C *IL-10* SNP associated with its lower expression, which could balance the IL-10 concentration. Analogical results were observed in an investigation that evaluated levels of IL-10 in patients with oral leukoplakia and healthy controls (P>0.05) (29). Likewise, another study did not find a different expression pattern for IL-10 between leukoplakia of the oral cavity compared with healthy gingiva (30).

IL-10 is an anti-inflammatory cytokine (31), which is considered a key regulator of immune responses, downregulating the pro-inflammatory cytokines such as tumor necrosis factor-alpha (TNF-α), IL-6, IL-1, IL-8, and IL-12 (32). Recently, an immunohistochemical analysis revealed a significantly elevated expression of IL-8 (stroma) and TNF-α (epithelium and stroma) in oral leukoplakia without dysplasia compared with the normal oral mucosa (P=0.022, 0.0017, and 0.047, respectively) (33). Another study found altered IL-6 levels in leukoplakia with coexisting periodontitis in comparison to healthy volunteers (P<0.001) (34).

We speculated that the inflammation in VFL cases analyzed in this systematic review could have increased the levels of pro-inflammatory cytokines. However, the expression of IL-10 remained stable and it contributed to an inflammatory profile of these patients because normal IL-10 concentrations cannot decrease high pro-inflammatory cytokines levels, leading to an imbalance in the inflammatory profile. The genotype-specific disturbances in the expression of pro- and anti-inflammatory interleukins have been shown to alter the functioning of the immune system (32). We suggest that future studies evaluate the pro- and anti-inflammatory cytokines to assess whether there is an imbalance between them in individuals with VFL.

The -309 T/G *Mdm2* SNP was not a risk factor for VFL development (OR=0.72, P=0.39). However, the levels of the Mdm2 protein were exacerbated in cases compared to healthy controls (P<0.01) (17). This polymorphism is localized in the promoter region and can rise Mdm2 concentration (35). Mdm2 controls p53, a tumor suppressor protein that acts in important processes such as DNA repair, cell cycle arrest, apoptosis, and aging (36). When the level of cellular stress rises, p53 increases via the post-translational mechanism, leading to cell cycle arrest or apoptosis. In the absence of cellular stress, p53 is controlled by Mdm2 in the cell, and there is a feedback mechanism between these proteins in which when one increases the other declines (36). Therefore, the -309 T/G *Mdm2* SNP and the overexpression of Mdm2 increase cancer risk and accelerate tumorigenesis (37). Altered levels of Mdm2 could be an alert for the possibility of a malignant transformation in VFL.

The CT genotype of the rs11886868 C/T *BCL11A* SNP and the GG genotype of the rs4671393 A/G *BCL11A* SNP increase the risk of VFL(OR=3.30, P=0.011; OR=3.02, P=0.041, respectively) (6). Moreover, the G allele of the rs4671393 A/G *BCL11A* SNP markedly raised the risk of VFL development (OR=1.75, P=0.002), and the levels of BCL11A were significantly exacerbated in subjects with VFL compared with the control group (P<0.01).

The rs11886868 C/T *BCL11A* and rs4671393 A/G *BCL11A* SNP are in intron 2 of the *BCL11A* gene and are associated with BCL11A production (38). This protein has been related to many diseases such as type II diabetes, intellectual disability, β-hemoglobinopathies, cancer, and hematological malignancies, but the mechanisms by which BCL11A is connected to these diseases are not yet completely understood (39). BCL11A is a reducer of fetal hemoglobin gene expression (38) and it remains active in adulthood (40). Individuals with the AG or GG genotypes of the rs4671393 A/G *BCL11A* SNP are more likely to have a high concentration of BCL11A (6), which leads to a low level of fetal hemoglobin (38), and consequently anemia.

A recent study showed that subjects with oral leukoplakia had significantly greater deficiencies of iron (P=0.032), vitamin B12 (P<0.001), folic acid (P<0.001), and hyperhomocysteinemia (P<0.001) compared with healthy volunteers (41). Perhaps, patients with VFL from the study by Zhou et al. (6) could have had the same deficiencies because they presented an overexpression of BCL11A (P<0.01), a suppressor of hemoglobin production, which can lead to anemia development. It suppresses the immune system, and as a result, individuals become more prone to develop diseases and lesions like VFL.

There was a lack of association between the rs14024 *CK-1* SNP and VFL, (AG, OR=1.82, P=0.12; GG, OR=1.88, P=0.11) (5). Cytokeratins are keratin proteins that are part of intermediate filaments frequently found in epithelial cells (42). Keratinocytes and immune cells control skin inflammatory and immune responses, producing cytokines, antimicrobial peptides, and expressing other proteins (42). CK-1 is associated with skin diseases and epithelial tissue damage (43,44), therefore it could also be associated with VFL, which causes epithelial tissue damage.

The LL genotype and the L allele of the (GT)n repeat polymorphisms in the *HO-1* gene were risk factors for VFL (OR=3.72, P=0.039; OR=1.9, P=0.006, respectively) (16). Likewise, the levels of HO-1 were significantly lower in subjects with VFL than in the control group (P<0.01). HO-1 is an enzyme involved in the production of free iron, carbon monoxide, and biliverdin, which is transformed into bilirubin (45), substances with an anti-inflammatory and anti-oxidative role (46).

The (GT)n repeat polymorphisms are in the promoter region of the *HO-1* gene on chromosome 22q12 and can affect the secretion of HO-1 (47). The S allele is classified as a short allele with ≤26 (GT)n repeats, while the L allele is classified as a long allele, having >26 (GT)n repeats (48). Longer repeats are linked to a reduction in HO-1 secretion and activity (46,49), while shorter repeats are related to elevated HO-1 activity (46,49).

The LL genotype and the L allele of the (GT)n repeat polymorphisms in the *HO-1* gene were risk factors for VFL development, and HO-1 concentrations were decreased in cases compared to controls (16), which indicates a lower production of anti-inflammatory and anti-oxidative substances, increasing the likelihood of developing diseases. The (GT)n repeat polymorphisms in *HO-1* have been associated with severe acute pancreatitis (49), encephalitis in HIV infection (50), pediatric non-alcoholic fatty liver disease (51), and cancer (52), and lower levels of HO-1 are linked to diabetic retinopathy (53) and peripheral artery diseases (54).

Overall, the studies analyzed in this systematic review had a low risk of bias according to the NOS criteria, indicating the good validity of the present results.

Based on our results, the first hypothesis that genetic polymorphisms are involved in VFL etiology was accepted. The second hypothesis that the protein levels of MDM2, BCL11A, and HO-1 were altered in VFL patients was also accepted.

These data can be extremely important in clinical practice because these SNPs and proteins could be powerful markers for diagnosis and treatment. Treatments for VFL include speech therapy, surgical techniques, vocal fold injection (55), and the use of drugs (56). However, there is no effective therapy yet (1), and more indicators for developing new treatment options are needed. The molecular markers evaluated in this study could be potential indicators for better treatment outcomes. Natural products and pharmacological medications targeting IL-10 (57), MDM2 (9), BCL11A (58), and HO-1 (59) have been shown to be effective in clinical and pre-clinical studies involving other diseases and may also be effective in treating patients with VFL and preventing the onset of cancer.

Further research in different ethnicities is required to confirm the involvement of these markers in VFL, as all studies included in this systematic review were performed in China. Although the evaluated genetic markers are present in other populations such as from Austria, United Kingdom, America, Turkey, India, Finland, France, Poland, Pakistan, Egypt, Tunisia, Thailand, Iran Spain, Brazil, and Mexico (28,50,60-74), to the best of our knowledge, there are no published studies on the involvement of genetic polymorphisms in patients with VFL from these countries.

### Conclusion

Most genetic polymorphisms analyzed in this systematic review were risk factors for VFL development, and most proteins were modified in VFL patients. New markers could lead to the development of effective therapies for this lesion, avoiding a malignant transformation.
